# An Inside Look into Biological Miniatures: Molecular Mechanisms of Viroids

**DOI:** 10.3390/ijms22062795

**Published:** 2021-03-10

**Authors:** Srividhya Venkataraman, Uzma Badar, Erum Shoeb, Ghyda Hashim, Mounir AbouHaidar, Kathleen Hefferon

**Affiliations:** Cell and System Biology, University of Toronto, Toronto, ON M5S 3B2, Canada; byokem@hotmail.com (S.V.); uzmabadar.ca@gmail.com (U.B.); erumsh@uok.edu.pk (E.S.); ghydaHashim@cmail.carlton.ca (G.H.); mounir.abouhaid@utoronto.ca (M.A.)

**Keywords:** viroids, replication, rolling circle, pathogenicity, trafficking, viroid-host interactions, gene silencing, evolution, classification, identification

## Abstract

Viroids are tiny single-stranded circular RNA pathogens that infect plants. Viroids do not encode any proteins, yet cause an assortment of symptoms. The following review describes viroid classification, molecular biology and spread. The review also discusses viroid pathogenesis, host interactions and detection. The review concludes with a description of future prospects in viroid research.

## 1. Introduction

Viroids are one of the most inscrutable single-stranded, structured, circular RNA pathogens of plants as well as the smallest infectious agents ever known [1,2]. Despite being incapable of coding for any proteins, viroids affect susceptible plant hosts with visually discernible symptoms resembling those induced by several plant viruses. Diener, 1967, 1971 [3,4] discovered and exemplified the Potato Spindle Tuber Viroid (PSTVd), the first viroid ever known. He coined the term “viroid” to represent this diminutive, naked, protein-free, circular RNA plant pathogen [4,5,6]. This conceptualization of the viroid was further substantiated by Sänger, 1972 [7] as well as Semancik and Weathers, 1972 [8], who identified the citrus exocortis viroid (CEVd) that is responsible for causing the citrus exocortis disease. Another viroid, the chrysanthemum stunt viroid (CSVd) is also one of the viroids initially identified [9,10]. Following the initial identification of the PSTVd, numerous other viroids have been discovered [11]. There are currently about 32 recognized species as well as eight putative species of viroids [12]. Some of the most recently identified viroids include the columnea latent viroid (CLVd) and the pepper chat fruit viroid (PCFVd) infecting pepper in Vietnam identified by RNA-seq [13].

Viroids are covalently closed circular RNA molecules with rigid rod-like secondary structure ranging in size between approximately 250 to 400 nucleotides [14,15]. Viroids undergo autonomous replication and occur as circular and linear forms containing a high degree of base pairing while parasitizing the host transcriptional machinery [2,15]. Viroids are totally dependent on host cellular RNA polymerases and processing enzymes for their replication. They are categorized into two families: the Avsunviroidae (type species, Avocado sunblotch viroid ASBVd) and the Pospiviroidae (type species, PSTVd) [16,17,18,19,20]. Unlike viruses, viroids do not require to be encapsidated for propagation among host plants. Viroid RNA moves from infected cells into adjacent cells via plasmodesmata and then through the phloem to distant sink organs of its host plant [18,21]. Transmittance of viroids occurs through grafting, mechanical injury, pollen, seed and/or insects [21].

Viroids cause mild to severe diseases in economically significant crops including both herbaceous and woody plants such as for instance, apple, avocado, coconut, grapevine, hop, peach, potato and tomato [2,22]. Symptoms induced by viroids range from mild to severe, some causing even debilitating, lethal infections. Symptoms depend to a large extent on the strain of the viroid (its nucleotide sequence), the nature of the susceptible host (genotype), environmental conditions as well as the type and the severity of the disease they induce [21]. Viroids elicit disease symptoms by interacting directly with host factors, although the precise molecular mechanisms involved are yet to be completely understood. Increasing evidence on the viroid-derived small RNAs and their involvement in RNA silencing pathways has shed light into the molecular machinery involved in viroid pathogenicity.

It is very intriguing that viroids are able to cause several destructive diseases in their host plants while being diminutive infectious molecules. This means that the limited genetic information that they carry is sufficient to induce systemic infection in susceptible plants [19]. Considering that they are non-coding RNAs, viroids rely on their own genome information, covalently closed circular RNA composition and structure imposed by their very nucleotide sequences to replicate and induce diseases in their respective host plants [23,24,25,26,27,28]. Thus, they can be considered as “abnormal” regulatory RNAs and not operative messenger RNAs [4]. The viroid disease symptoms result from a complex interaction between the pathogenic viroid RNA and the plant genome and the ensuing changes within the host cellular machinery that translate into alterations of the physiology of the host plant [20].

Viroids negatively impact the world’s economy by causing the destruction of susceptible crop plants and cause unbridled spread of infection leading to several important plant diseases. A majority of the crop devastation associated with infection by plant viruses [29] largely applies to viroids. Hence, there is a compelling need to study the molecular biology of viroids as well as their complex interactions with their hosts and the insect species which are involved in their transmission to design viable intervention strategies towards improving crop security.

With biotechnological advancements such as microarrays [30] and next-generation sequencing technologies (NGS) [13], novel viroids, their variants and novel host-pathogen interactions are being identified in diverse geographical regions. For instance, RNA-seq technology and computational algorithms have been employed to discover the grapevine latent viroid (GLVd) and the grapevine hammerhead viroid-like RNA (GHVd-like RNA) in grapevines and deep sequencing has been useful in identifying the apple hammerhead viroid-like RNA (AHVd-like RNA) in apples [31,32]. Additionally, grapevines in India have been recently shown to host viroids such as Australian grapevine viroid (AGVd), hop stunt viroid (HSVd) and the grapevine yellow speckle viroid 1 (GYSVd-1) indicating wide geographical distribution of viroids [33,34]. NGS studies have already been executed for some members of the Pospiviroidae family such as citrus bark cracking viroid (CBCVd) [35], HSVd [36], hop latent viroid (HLVd) [37] and the PSTVd [38].

Several contemporary reviews have addressed many of the viroid characteristics such as their structure, replication, trafficking and disease determining modes in host plants [15,20,21,22,39,40,41]. Over the last decade, many technological approaches like biochemistry, molecular biology, computational biology and modern sequencing technologies are increasingly being employed to understand viroid biology, viroid pathogenicity and viroid-host interactions. However, a comprehensive summary of current literature elucidating viroid molecular biology, pathogenicity, trafficking, transmission strategies, viroid–insect–plant interactions and viroid detection technologies in the light of the recent technological breakthroughs is lacking. Therefore, the major objective of this review is to highlight the above molecular characteristics of these biological miniatures in the context of the latest biotechnological advancements. Finally, the review closes with an insightful discussion regarding the taxonomy and evolution of viroids in the light of recent discoveries in the viroid field. This review is only focused on discussing the molecular mechanisms involved in viroid infections and does not address diseases caused by viroids which could be a subject, albeit a vast one, for another review.

## 2. Biology of Viroids

Discovered in 1971, viroids are the smallest and simplest plant pathogens, ranging from 250 to 400 nucleotides of RNA, and are known as “living fossils of the hypothetical RNA World” [42]. These autonomous infectious agents are devoid of any protein coding capability and can survive without any protected membrane around their single-stranded, circular RNA genomes. Viroids can not only fight the host cellular mechanisms of RNA degradation but can also successfully replicate themselves using factors from the host plant, resulting in their spread throughout the plants and disease symptoms [14]. Angiosperms are reported as the only natural hosts of viroids [43].

The processes involved in viroid infection after entry into cells includes [15,24,27,44].

(a) entry into a subcellular organelle (chloroplast or nucleus according to the type of viroid),
(b) rolling circle replication within chloroplast or nucleus,
(c) release out of the cell following replication,
(d) transport into nearby cells,
(e) entry into and within the phloem,
(f) invasion of nonvascular cells from the phloem and,
(g) repeat of the infection cycle.

The viroid genome does not encode for any protein, but instead spreads throughout the plant by recruiting host proteins using their own RNA folded structure. Functional motifs within the RNA structure are required for cellular trafficking in host plants [45]. The nucleotide sequence of the viroid is also important for interacting with the genome of the host. RNA-directed DNA methylation (RdDM) has been reported in viroid-infected plants [46,47] and examples of silencing of functional genes by individual viroid derived small RNA (vd-sRNA) have been reported [48,49,50].

Viroids replicate in host plants using host RNA polymerases that interact with the viroid RNA template, resulting in a higher than usual rate of errors in comparison to DNA replication. Hence, a given replicating viroid produces several mutants along with its original sequence, the mix being termed quasispecies [51,52]. These closely related quasispecies exhibit vast variation in their host invading capability, demonstrating that the property of infecting the host depends mainly on nucleotide sequence of the viroids. Furthermore, the cause of variability in symptoms of viroid infections also depends on viroid-host-environment interactions.

The cytopathology of family Pospiviroidae include cavity formation within the cell membrane and cell wall thickening [53]. Peach latent mosaic viroid (PLMVd) infections were demonstrated to lead to chloroplast malformations [54]. Tracking viroid cytopathic effects represents a promising approach to understand the links between disturbances at the cellular level and macroscopic symptoms [55,56].

Viroids are distinguished on the basis of their transmission within host plants following either vertical or horizontal patterns [57]. Pollen and ovules of a plant are responsible for transmission of viroid infections from parental plants through seeds and then seedlings to the next generation in the vertical mode of transmission; an example is the PSTVd. During horizontal transmission (an example is Tomato planta macho viroid), infection is transmitted to the next generation through the ovaries of a plant getting infected from physical contact of another plant without fertilization [58]. The degree of vertical and/or horizontal transmission of viroids depends on the molecular interaction of the viroid with the host plant, resulting in recognition and elimination of viroid RNA in the male gametophyte [59].

Mediator (MED) is a conserved protein complex in plants. According to recent reports, MED subunits exhibited differential expression patterns against different viroids, suggesting that the connection of the MED subunit transcriptional reprogramming with viroid infections resulted in differences in symptom development for different viroids [60].

## 3. Taxonomy and Classification of Viroids

According to the International Committee on Virus Taxonomy (ICTV), “rules concerned with the classification of viruses shall also apply to the classification of viroids” [61].

Viroid classification is based on secondary structure. Currently 32 species of viroids are recognized by ICTV. The observation of quasispecies poses an important taxonomic question for considering related viroid variants as of the same or different species. Less than 90% genome sequence identity was the first criterion taken into consideration and to be accompanied later by the possession of another distinguishing biological feature, in order to classify any two viroid species as distinct, as per the ICTV classification requirements [17]. Table 1 highlights the molecular criteria that are considered in the classification of viroids [2].

Viroids are divided into two families: Avsunviroidae and Pospiviroidae [2,15,24,43,44,62,63] (Table 2). Family Avsunviroidae contain three genera with members resembling ASBVd with branched secondary structures chloroplast-based symmetric rolling-circle replication mechanism and most importantly having ribozyme activities. On the other hand, family Pospiviroidae with five genera has PSTVd-like members, consisting of five major domains as secondary structures, asymmetric rolling-circle replication that occurs in the nucleus and are commonly devoid of any ribozyme activities. Avsunviroidae infect only dicotyledonous plants, both herbaceous and woody [20] while viroids of the family Pospiviroidae can infect both monocot and dicot plants [64].

The three genera of family Avsunviroidae are: Avsunviroid, Pelamoviroid, and Elaviroid. Species in the genus Avsunviroid, have a low content of G + C with rod-like secondary structure. Species of genus pelamoviroids have high G + C content with branched secondary structures. The single species of genus elaviroid have intermediate structural properties that are between those of the other two genera of the family [56].

**Figure 1:**
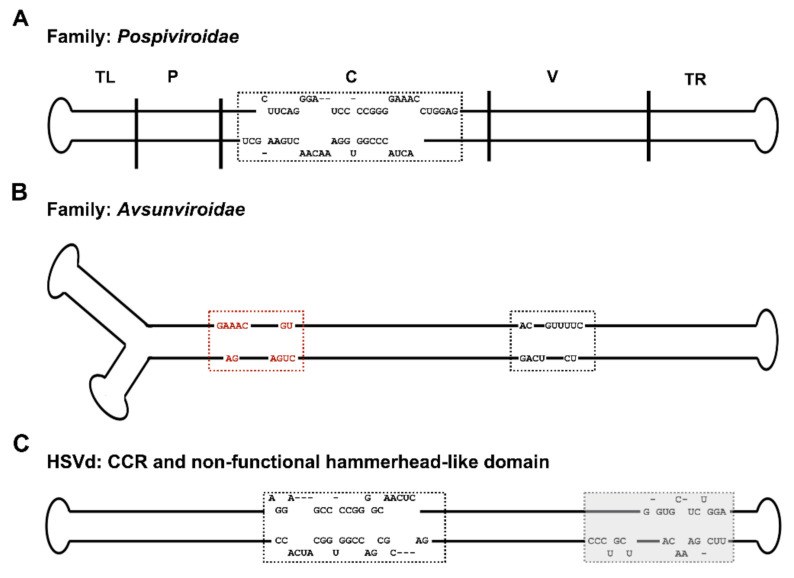
Structural differences among viroids. (Adapted from Adkar-Purushothama and Perreault, 2020 [66]). Primary and secondary structural characteristics considered for classification of viroids: (**A**) the PSTVd rod-like secondary structure comprising five functional domains is shown: the Terminal left (TL), Pathogenicity (P), Central (C), Variable (V) and Terminal right (TR) domains are separated by solid vertical lines. The nucleotide sequence of the central conserved region (CCR), a typical feature of the family, Pospiviroidae is shown within the box. (**B**) The ASBVd branched secondary structure is shown. Boxes represent the conserved nucleotides of the catalytic core within the hammerhead, a typical feature of the Avsunviroidae. The red and black boxes, respectively, indicate the hammerhead self-cleaving structures formed within the (+) and (−) strands. (**C**) The HSVd rod-like secondary structure is shown. The black-colored box represents the CCR while the shadowed box represents the non-functional hammerhead-like domain.

Five genera of family Pospiviroidae are: Apscaviroid, Cocadviroid, Coleviroid, Hostuviroid and Pospiviroid. Each genus has a characteristic central conserved region (CCR) with least modifications, while the species of genera apscaviroids and pospiviroids have terminal conserved regions (TCR) and those of cocadviroids and hostuviroids have terminal conserved hairpins (TCH). The Coleviroid species are devoid of TCR and TCH [65]. Figure 1 exemplifies the various structures adopted by these viroids [66].

## 4. Viroid Structure and Replication

PSTVd was the first viroid structure ever determined [4]; electron microscopy revealed that PSTV forms a secondary structure, and this was confirmed in 1978 [67,68].

Most of the known viroid species belong to the *Pospiviroidae* family, named after the type member, the PSTVd [15]. The Pospiviroidae adopt circular, internally base-paired, rod-like structures and their genomes consist of five distinct domains: The terminal left (TL), terminal right (TR), central (C), pathogenic (P) and variable (V) domains. The pathogenicity domain possesses relatively low thermodynamic stability (or pre-melting) due to the presence of an oligopyrimidine stretch in most of the *Pospiviroidae*, the V domain is the most variable region, and the C domain is most highly conserved.

To date, only four viroids have been identified as belonging to the *Avsunviroidae* family, named after ASBVd. These types of viroids lack a conserved central region but retain rod-like and branched regions like Pospiviroidae viroids. However, unlike Pospiviroidae viroids, *Avsunviriodae* members can form hammerhead ribozyme motifs in both polarities which mediate cleavage of their replication intermediates, while RNA cleavage within *Pospiviroidae* family members takes place using host enzymes [15].

**Figure 2:**
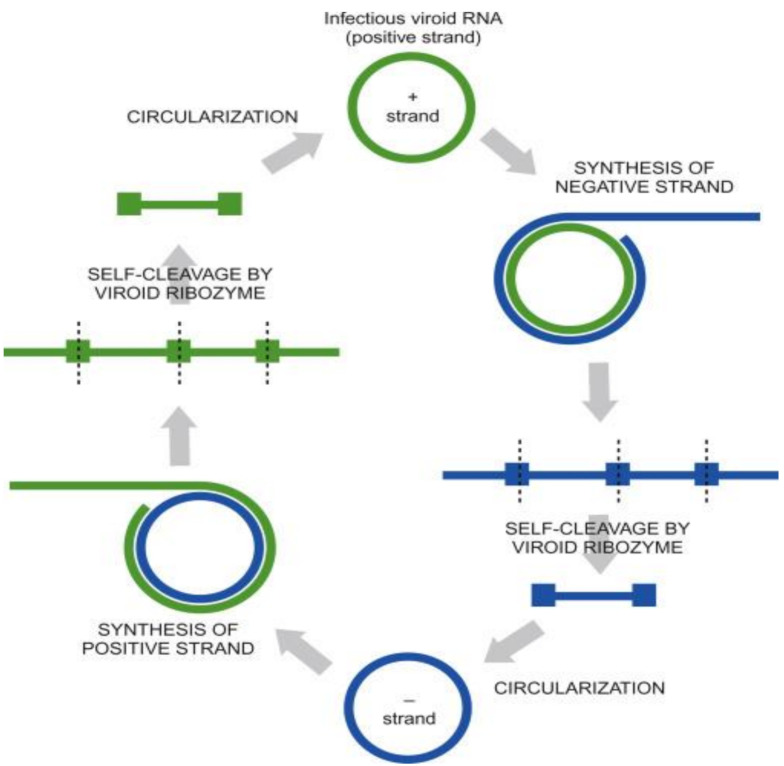
Rolling circle model for Avsunviroid replication (adapted from Clark et al., 2019 [69]).

The replicative mechanism of viroids operates through two alternative pathways depending in whether it is mediated by one or two rolling circles [21]. The first pathway is called asymmetric, typical of Pospiviroidae and is mediated by a single rolling circle wherein the incoming circular positive sense viroid RNA genome is repeatedly transcribed to form multimeric, linear (−) strands. Thereupon, in the next step, these (−) sense concatemers serve as templates for the synthesis of multimeric, linear (+) strand concatemers which subsequently are cleaved into monomeric (+) circles. In the alternate second pathway called symmetric, typical of Avsunviroidae (Figure 2) [69], replication operates through two rolling circles wherein the multimeric linear (−) strands generated from the (+) sense RNA genome of the viroid undergo cleavage and ligation to generate circular (−) strand RNA monomers. These (−) strand circles then act as templates for the subsequent generation of linear, multimeric (+) strands which then cleave into (+) sense monomer genomes.

For PSTVd, the type member of Pospiviroidae, the absence of circular monomeric (−) sense RNA in plants after natural infection supports the asymmetric model of replication [70,71]. On the other hand, for the ASBVd (the type member of Avsunviroidae) it has been shown by in vitro studies that both (+) and (−) dimeric RNAs are able to self-cleave (Figure 2), thus supporting the model of symmetric replication [72,73]. Additionally, it has been observed in ASBVd-infected avocado that both monomeric circular (+) and (−) strands occur in multistranded complexes substantiating that ASBVd replication occurs through two rolling circles [74,75].

Members of the *Pospiviriodae* replicate in the nucleus [76]. Replication is initiated from a precise site, thus implying the possibility of existing viroid promoters. The processes of cleavage and ligation in the *Pospiviroidae* family are thought to be catalyzed by a host enzyme similar to RNase III and an RNA ligase that supports the circularization process. The two enzymes can circularize the viroid by covalent fusion of both 5′ and 3′ termini. Researchers have not identified whether chloroplastic RNA ligase is necessary for the circulation process or whether autocatalysis takes place.

*Avsunviroidae* replication takes place within chloroplasts. The mode of entry and exit into the chloroplasts is still debatable as it is not well identified [76].

The accumulation of *Avsunviriodae* (+) and (−) strands in the chloroplast indicates the involvement of enzymatic machinery of the chloroplast in the replication cycle; in contrast, accumulation of the *Pospiviroidea* RNA strands in the nucleus suggests the involvement of nuclear RNA polymerase and other cellular enzymes in their replication cycle [21].

## 5. Movement and Systemic Trafficking of Viroid RNAs

Viroids are subviral pathogens that cause infection in several crop plants, leading to considerable yield losses [77]. Within the plants, viroid RNA moves to adjacent cells through plasmodesmata and, via the phloem, to distant sink organs [23]. Viroids have recently emerged as ideal model systems to study RNA transport within and between cells [23]. Conventional viroid infection of a host plant comprises a series of coordinated steps that involve both intracellular movement and intercellular movement.

Mutational experiments of viroids have identified RNA motifs within the viroid genome that are important for cell-to-cell trafficking. For PSTVd, this consists of 27 RNA loop motifs separated by short helices [74,78,79]. An RNA motif of PSTVd was found to be essential for trafficking from bundle-sheath cells into mesophyll cells when the viroid was exiting in the phloem of young tobacco leaves [26]. Whereas in *Nicotiana benthamiana*, a different RNA motif was required for movement of PSTVd from the bundle sheath cells into the phloem [80]. Furthermore, 11 out of 27 loops of PSTVd RNA motifs are important for cell-to-cell movement and intercellular spread and these RNA motifs could also be involved in the trafficking of viral and cellular RNAs [81]. Loop 19 was identified for viroid movement from palisade to spongy mesophyll cell of *N. benthamiana* [82], while loop 6 had previously been shown to be essential for palisade-to-spongy mesophyll trafficking [83]. These studies enlighten the potential functions of plasmodesmata (PD), as different RNA motifs are required to transit PD at different cell-to-cell interfaces. It was also identified that different RNA motifs can be used to transit across the same cellular interface.

Various experiments have been performed to identify viroid RNA movement. Microinjection experiments using infectious RNA transcripts and labeled with the fluorescent dye TOTO-1 iodide have shown that PSTVd can move rapidly from cell to cell via the plasmodesmata in tobacco mesophyll cells [84]. It was also observed that PSTVd RNA accumulates in the nuclei of both the injected cell and neighboring cells. Using dot-blot hybridization to monitor PSTVd distribution in infected tomato seedlings, it was found that the movement pattern of PSTVd was indistinguishable from that of most plant viruses at the whole plant level [84].

## 6. Seed, Pollen and Insect Transmission of Viroids

Most of the viroids are disseminated through human activities during planting and through trade in materials of the plants, such as seeds and tissue culture stocks, but some viroids have evolved specific mechanisms exploiting the plants′ processes of reproduction and are transmitted via seed and/or pollen [85,86,87]. Pollen is an important breeding biological tool of germplasm naturally found to be associated with number of viroids, known as being pollen-transmitted [88]. Transmission of viroids by pollen can be horizontal, contaminating a fertilized flower, or can be vertical which is more common when infected pollen fertilize and infect the resulting seed [89]. Pollen transmitted PSTVd and PCFVd have been detected in tomato (*Solanum lycopersicum*) crops [90].

The rate at which infected seeds produce infected plants is called the seed transmission rate. The lower rate of seed-transmission in PSTVd could be due to the restricted movement of viroids in floral organs. In *Nicotiana benthamiana* and *Solanum lycopersicum,* the accumulation of PSTVd was not observed in the petals, ovary and stamens, but detected only in the sepals [91]. In petunia, PSTVd is seed-transmitted either through viroid-carrying pollen grains or embryo sacs.

Tomato chlorotic dwarf viroid (TCDVd) of the genus Pospiviroid infects *Solanum lycopersicum* and is distributed in the ovary and ovules but not in the shoot apical meristem [92,93]. Matsushita et al. [93] reported that TCDVd was found on the surface of the seed coat.

In *Capsicum annuum L*. PCFVd, another Pospiviroid, was reported to be seed-transmitted [94]. The presence of the Pospiviroids GYSVd 1 and HSVd were found in *Vitis vinifera* seedlings [95].

ASBVd is an example of Avsunviroid transmitted from infected trees to the seeds of the next generation [96,97,98]. Another species of family Avsunviroidae genus Pelamoviroid, PLMVd, is not reported to be pollen transmitted and is not seed-transmitted [99,100,101]. The only known species of genus Elaviroid in the family Avsunviroidae Eggplant latent viroid (ELVd), which is reported to be seed-transmitted via eggplants [102,103].

Additionally, insect-transmitted mechanical inoculation of viroids was demonstrated to be a source of infection through plants via pollen [104]. Insect pests are known for their tendency to transmit PSTVd to *Solanum tuberosum* (potato) [105]. *Myzus persicae*, commonly known as green peach, aphid-transmitted PSTVd from other plants coinfected with viruses [106,107,108]. Apple scar skin viroid (ASSVd) was found to be transmitted by *Trialeurodes vaporariorum* (whitefly) from viroid-infected plants to cucumber, bean, tomato and pea plants [109].

## 7. Pathogenicity of Viroids

The first complete sequence of the PSTVd was reported by Gross et al. [67]. However, it was assumed that viroid pathogenicity was due to direct interaction with one or more of the host cellular constituents and or by indirect interaction via RNA silencing. Viroid pathogenesis has been shown to be increased due to the interaction with nuclear and cytoplasmic RNAs which results in the activation of protein kinases. It has been reported by Hiddinga et al. [110] that a 68-kDa protein extracted from viroid infected tissues is differentially phosphorylated, and that dsRNA dependent protein kinases similar to their equivalents in mammalian cells are involved in the regulation of viroid synthesis. In this case, the binding of protein kinases enhanced viroid pathogenicity. The PSTVd strains produced different symptoms with only 3–4 nucleotides changed [111] and this change could alter the protein binding site resulting in abnormal function [112]. Infectious cDNA clones were first constructed for PSTVd [113] and then for other viroids to determine their pathogenic determinants [114,115,116]. It is also noticeable that in some cases, viroid accumulation to high titers is observed although the plants are asymptomatic or while on the contrary, other viroids at low titers cause severe symptoms, which represents the contribution of alternative mechanisms [117].

At the microscopic level, the cytopathogenic effects of viroids on host cellular structures have been reported. For example, for some viroid infections, there is an abnormal development of cytoplasmic membranes to form “plasmalemmasomes”, irregular thickening of cell walls [118], chloroplast abnormalities and electron-dense deposits in the cytoplasm and chloroplasts [55].

In recent studies, various molecular mechanisms are involved in the induction of viroid diseases and in this context, RNA silencing has a crucial role in viroid pathogenesis. RNA silencing was first identified in plants and then in other eukaryotes, where it provides other novel regulatory roles in addition to translation. There is very strong evidence that the combined action of the structure-specific dicer-like proteins and the sequence-specific RNA induced silencing complex curb plant RNA and DNA virus infections as well as viroid infections [36,38,119,120,121,122]

## 8. Viroid-Host Interactions

Being naked, noncoding RNA molecules, viroids induce disease through direct interaction of their genome with some of the host factors. Despite their simplicity in both genome and structure, viroids elicit complex responses in their host plants wherein even with minor changes in their nucleotide sequence, they can induce entirely different symptoms in their host plants based on the cultivar [35]. In general, all viroids elicit “pathogenesis-related” [PR] proteins during their infection cycle [37].

Earlier, the PSTVd was shown to associate with several nuclear and histone proteins of tomato [123]. Initial investigations of the leaf proteins of tomato plants infected with PSTVd revealed a significant increase in the levels of the PR protein, P14 [124]. Introspection into the expression of genes involved in stress response, defense response, chloroplast function and cell wall structure revealed altered expression depending on severity of PSTVd infection [125,126]. In another study, the wheat germ RNA polymerase II was shown to interact with terminal loops of the PSTVd [127]. Martínez de Alba et al. [128], demonstrated that the tomato Virp1 bromodomain-containing protein bound to the PSTVd 71-nucleotide bulged TR hairpin structure and that this association played a significant role in systemic spread of the PSTVd [129]. The tomato protein, p68 involved with the ds-RNA-induced protein kinase activity was shown to be differentially phosphorylated by the PSTVd [130,131]. Moreover, the PSTVd was demonstrated to differentially activate the p68 protein based on the severity of the viroid strain, thus implicating the p68 protein in viroid pathogenesis. Interestingly, this differential p68 activation was detected with strains of PSTVd that varied only by a single two-nucleotide inversion within the lower pathogenicity domain of the PSTVd which caused minor variations in their secondary structures. Hammond and Zhao, [132] reported increased transcription of a novel protein kinase (PKV protein) in tomatoes infected by PSTVd depending on the severity of infection. Additionally, they also showed down-regulation of genes responsible for chloroplast biogenesis as well as impacts on the mRNA levels involved in gibberellin biosynthesis and those of some of the signaling hormones. The PSTVd replicates in the nucleus for which the replication start site is present within the hairpin loop of the TL region of its secondary structure and transcription is mediated by the host DNA-dependent RNA polymerase II [133]. In Arabidopsis thaliana, the transcription factor IIIA [TFIIIA] and the ribosomal protein L5 [RPL5] have been shown to play a role in PSTVd replication by binding to the (+) strand of the viroid [134]. Additionally, in Nicotiana benthamiana, the canonical 9-zinc finger [ZF] Transcription Factor IIIA [TFIIIA-9ZF] as well as its variant TFIIIA-7ZF were demonstrated to interact with the PSTVd (+) strand while only the latter recognized the PSTVd (−) strand. Plus, the expression levels of TFIIIA-7ZF directly correlated with viroid replication [135]. PSTVd reportedly recruits the RPL5 splicing regulator to interact with the CCR that plays a critical role in its replication [136,137].

The *Cucumis sativus* Phloem Protein 2 RNA binding protein [CsPP2] was shown to be associated with long distance movement of HSVd RNA by forming a ribonucleoprotein complex [138,139]. The CsPP2 was also demonstrated to enhance the efficiency of transfer of the ASSVd through the *Trialeurodes vaporariorum* [Tv] Whitefly [109]. To-date, the Virp1 and CsPP2 proteins are the best elucidated factors shown to be involved in translocation of the Pospiviroidae. Among Avsunviroidae, the ASBVd infection in avocado revealed the involvement of PARBP33 and PARBP35 chloroplast RNA-binding proteins with the self-cleavage of ASBVd multimer transcripts mediated through hammerhead ribozyme [140].

Tomato plants infected with CEVd showed changes in levels of proteins involved in translation [141]. Additionally, CEVd reportedly induced as well as reduced in vitro phosphorylation of a wide range of proteins when infecting its host plants particularly, at the beginning of symptom appearance [142]. These changes in phosphorylation were enhanced in the presence of Mn2^+^, demonstrating the significance of Mn2^+^-dependent protein kinase action on the varied phosphorylation patterns. In this context, Hidding et al., 1988 [110] reported enhancement in the phosphorylation of a 68 kDa host protein homologous to the human ds-RNA-dependent protein kinase in tomato plants infected with PSTVd. Cottilli et al., 2019 [143] demonstrated changes in the translational machinery of tomato plants infected with the CEVd. They detected the presence of the CEVd within the ribosomal fractions and the CEVd impacted the polysome profiles, specifically causing the accumulation of the 40S ribosomal subunit. The CEVd was also shown to alter ribosome biogenesis and 18S rRNA maturation. Further, the levels of the ribosomal stress mediator NAC082 was increased in infected leaves. These changes correlated with the extent of disease symptoms caused by CEVd. Therefore, these findings showed that in tomato plants CEVd causes defective ribosome biogenesis and impacts the machinery of translation resulting in ribosomal stress.

Viroids, even without having any ability to code for proteins, impact the translational machinery. The CEVd has been demonstrated to cause changes in the accretion of ribosomal proteins such as S3, S5 and L10 in tomato plants [141]. It also impacts the levels of the eEF1A, eEF2 and eIF5A eukaryotic translation factors in these plants. Certain viroids have been reported to associate with the eIF1A or with the L5 ribosomal protein [134,141,144]. The HSVd elicits alterations in the DNA methylation patterns of the rRNA genes in host plants and results in increased accumulation of some of the rRNA-derived sRNAs [145]. HSVd caused demethylation of some of the rRNA genes leading to transcriptional reactivation of these genes, suggesting a novel molecular mechanism putatively involved in viroid pathogenicity. Moreover, it has been shown that the PSTVd induces degradation of ribosomal protein S3a-like mRNAs in infected tomato plants [146].

Further, an augmented number of differentially regulated genes was observed in peach plants doubly infected with both PLMVd and Prunus necrotic ringspot virus (PNRSV) when compared to those of single infections with either of the two viroids. The double infection also caused a synergistic impact on the peach fruit transcriptome [147]. The PLMVd (that replicates in chloroplasts) upon infection of *Prunus persica* (peach) induced the expression of six potential RNA-binding polypeptides, one of which is the elongation factor 1-alpha [eEF1A].

## 9. Impact of Viroid Infection on Gene Silencing and Indirect Influence of Viroids on Host Genes and miRNAs

**Figure 3:**
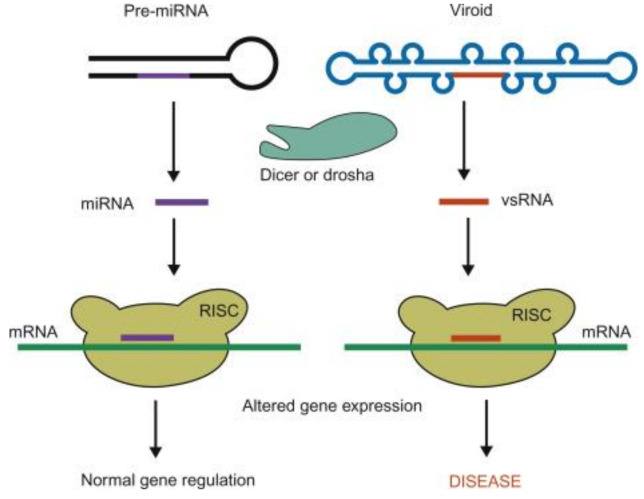
Viroids induce disease by generating viroid small RNA (vsRNA) that elicit RNA interference. (Adapted from Clark et al., 2019 [69]).

Being noncoding RNAs, viroids do not possess silencing suppressor activities found in viruses [148]. However, several research studies reported the accumulation of 21–24 nucleotide-long viroid-derived small RNAs [vd-sRNAs] in viroid-infected plants. Generation of vd-sRNAs impacts disease symptom expression in host plants (Figure 3 [69]). This induction of vd-sRNAs was observed irrespective of the site of viroid replication and showed that viroids can induce RNA silencing of host genes [149,150,151]. This induction of the vd-sRNA was observed in response to both (+) and (−) strands of the viroid RNA, although the genomic (+) strands induced more vd-sRNA than the antigenomic (−) strands as detected by next-generation sequencing [152,153,154]. The difference in vd-sRNA accumulation could be due to the lesser levels of accumulation of the (−) strands compared to that of the (+) strands as the former only act as replication intermediates [73,155,156]. However, the PSTVd replication was found to be resistant to RNA silencing and could be so by virtue of its inherent secondary structure that renders it recalcitrant to RISC-mediated cleavage. The occurrence of vd-sRNA hot spots in viroids reveals the presence of RNA silencing susceptible regions within the secondary structure of the viroids [157]. Deep sequencing of the vd-sRNA from tomato plants [cv. Rutgers] infected with PSTVd showed that most of the vd-sRNAs generated were derived from the PSTVd pathogenicity-modulating domain [49]. PSTVd mutants unable to target host callose synthase mRNAs failed to induce disease symptoms. In PSTVd-infected tomatoes, using algorithms for computer-based target prediction, Adkar-Purushothama and Perreault, 2018 reported a single vd-sRNA capable of promoting RNA silencing of more than one host gene involved in defense mechanisms [158].

**Figure 4:**
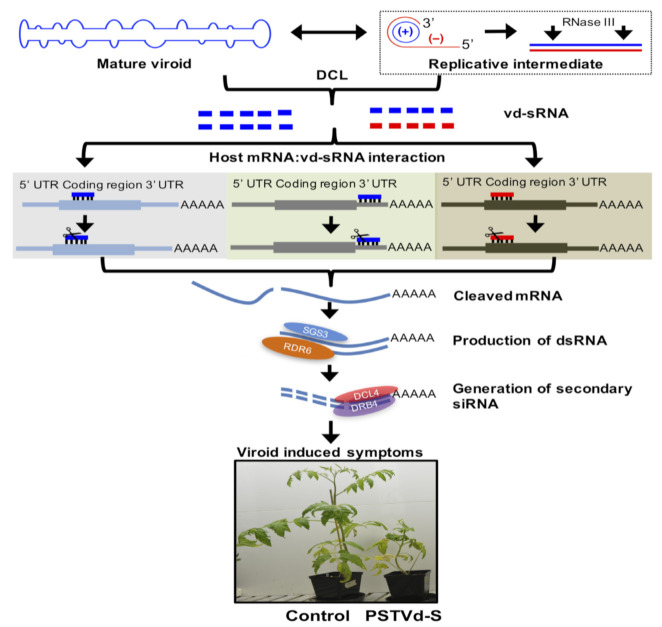
Mechanism of viroid symptom induction. (Adapted from Adkar-Purushotama and Perreault, 2019 [2]).

Equivalent findings were observed with the Avsunviroid PLMVd [159]. Malfitano et al. [116] reported a PLMVd variant containing a specific 12–13-nt hairpin insertion that causes albinism in susceptible plants. This sequence-dependent induction of disease symptoms is associated with the ability of the PLMVd to targetedly cleave the mRNA that codes for the chloroplast heat-shock protein 90 as evident from deep sequencing, rapid amplification of cDNA ends (RACE) analysis and semi-quantitative reverse transcription-polymerase chain reaction (RT-PCR). This implicates the involvement of RNA silencing in symptom expression induced by viroids (Figure 4 [2]). Besides, several other studies showed the role of vd-sRNAs in downregulating host genes [160,161,162].

PSTVd-infected plants compromised for the host RNA-dependent RNA [RdR] polymerase-6 showed enhanced amounts of both vd-sRNAs and PSTVd [163]. In another study, RdR6-silenced line of N. benthamiana plants infected with HSVd showed symptoms in stock plants whereas its scions were bereft of symptoms despite the accumulation of HSVd [164]. Viroid infection induces the generation of secondary siRNAs capable of impacting host mRNAs that are not the direct targets of vd-sRNAs. This phenomenon induced widespread cleavage and destruction of the host plant mRNAs. Earlier studies reported that as part of an antiviroid defense process, the RdR1 polymerase of the host tomato plants was triggered upon PSTVd infection [165]. Additionally, host plant miRNAs involved in leaf development were shown to be accumulated in tomato plants infected with PSTVd. Tsushima et al. [166] used deep sequencing to demonstrate changes in the profile of miRNAs involved in functions such as leaf curling, leaf development and disease resistance in PSTVd-infected tomato plants.

## 10. Viroid Detection and Identification

Viroids can be identified using various techniques. A popular viroid identification method uses biological indexing of a specimen plant on a variety of indicator hosts [21]. The indicator host shows diagnostic symptoms if infected by a particular pathogenic viroid. For better results of biological indexing, a researcher should be able to identify both the host range and the symptoms produced on the host by a specific viroid. Therefore, prior knowledge of the indicator host is necessary before attempting to identify any viroid. Several analytical assays such as RT-PCR, polyacrylamide gel electrophoresis (PAGE) and RT-Loop mediated isothermal AMPlification (RT-LAMP) have been applied for higher-scale sample testing and viroid indexing [21]. Nevertheless, these molecular techniques cannot detect the biological characteristics of the viroids such as their pathogenicity and infectivity. It is therefore evident that biological assays are required to bridge the gap.

There are levels of complexity in viroid identification that depend on knowledge of their molecular properties [21]. The traditional methods of viroid detection involve an assortment of native and denaturing polyacrylamide gel electrophoresis; the method relies on some properties of the viroid RNA such as its circular form to distinguish it from other RNAs. These older methods are still applied to identify the presence of new viroids; however, they cannot be used to identify each specific viroid, since the electrophoretic movement may be common over a wide range.

Viroid detection can also be performed using molecular hybridization. Owens et al. [167] were the first to show how this method can be applied for viroid detection. The technique is useful for detecting viroids from a known sequence; therefore, it relies on the availability of sequence information and is not suitable for the identification of new viroids. On the other hand, RT-PCR can be used for detecting several different viroids and has proven useful in identifying carrier plants that do not show any symptoms. For instance, some researchers used Pospiviroid primers based on a specific sequence to identify several viroids in a natural infection of tomato. A few viroids had not been earlier reported to be found in this crop, and research conducted by Verhoeven et al. [94] demonstrated that this method could be used for detecting new viroids. Additionally, this method is more reliable than those earlier discussed and hence is most used for the detection of viroids. However, the method is extremely sensitive and depends on the knowledge of nucleotide sequence; it is also affected by even a single mutation, which may limit the number of strains detected.

Among the various plant pathogens, viroids are the pathogenic organisms detected for the first-time using RT-PCR [168]. Subsequently, various amplification tools such as real-time RT-PCR [169], RT-PCR- Enzyme-Linked Immuno Sorbent Assay (ELISA) [170], (RT-LAMP) [171], multiplex bead-based array [172], multiplex RT-PCR [173,174] and in situ RT-PCR have been applied for the detection and identification of viroids [175].

End point RT-PCR has been applied for viroid detection in one- [176,177,178] or two-step reactions [168]. Additionally, polyvalent identification has been employed by multiplex amplification using a mix of specific sets of primers [173,174,179,180]. Results for this method can be observed through gel electrophoresis or by fluorescent [181] and standard or multiplex RT-PCR-ELISA [170,182]. Boubourakas et al. 2011 [175] adopted a modified RT-PCR technique using SYBR Green for in situ localization and detection of PLMVd.

Real-time RT-PCR has been used for viroid detection, assessment of plant resistance, viroid transmission and genotyping [169,183,184,185,186,187,188]. Additionally, qRT-PCR helps in quantitating the amounts of the concerned viroids.

Notomi et al. 2000 [189] first formulated the Loop-mediated isothermal AMPlification (LAMP) technique in which at least four primers specifically capable of recognizing six distinct nucleotide sequences on the target template under isothermal conditions (60–65 °C) [189,190] were used. Prior to the LAMP amplification procedure, RT reaction was carried out separately [191,192] or along with the LAMP reaction [193,194] using reverse transcriptase enzymes that can withstand 60–65 °C reaction temperature. The LAMP technique is based on the DNA strand-displacement activity of the Bst DNA polymerase and the reaction components include dNTPs in high amounts, magnesium sulfate and betaine used to diminish base stacking and to enhance target sequence selectivity [195]. The end products of LAMP are obtained as DNAs that are double-stranded stem-loops containing many inverted repeats of the target sequence as well as multiple loops forming cauliflower-like structures [189,190]. Therefore, the RT-LAMP reaction generates high DNA yield and the increased quantity of the produced by-product magnesium pyrophosphate enables direct visualization as a white-colored precipitate in positive samples [194]. Additionally, end point measurements or real-time turbidity assessments can be assayed at 400 nm [192,193]. Upon gel electrophoresis, the RT-LAMP reaction products appear ladder-like because of the cauliflower-like structures [196]. Thus, RT-LAMP technique by virtue of its simplicity, ease of use and power of detection helps in translating research in the laboratory to the field level. Table 3 lists some of the viroids for which RT-LAMP procedures have been adopted.

Microarrays serve as an optimal platform for one-step diagnosis of multiple viroid infections [30]. The numerous viroid nucleotide sequences obtained from public databases can be used to generate microarrays that enable detection of all of these viroids simultaneously. Additionally, related viroids can be identified by their unique hybridization patterns. Further, microarrays can be used for discovering new viroids by incorporating microarray elements derived from highly conserved sequences of viroid families, genera or species, particularly in cases involving plant diseases of unknown etiology.

The advantage of microarrays lies in their ability to detect a wide range of viroids using just a single test in addition to enabling highly specific oligonucleotide-based identification and ease of in silico design. Thus, mixed infections due to various viroids as well as related strains can be identified. However, the main caveat of this method is its complex and expensive detection process. Microarrays have been used in the identification of Pospiviroidae such as Tomato apical stunt viroid (TASVd) in tomato [202,203], PSTVd in potato [203,204], ASSVd in apples [203] and Citrus dwarfing viroid (CDVd) in citrus [203] as well as Avsunviroidae such as ASBVd in avocado [203], CChMVd in chrysanthemum [203] and PLMVd in peach [203].

NGS biotechnologies and bioinformatics were initially used for the detection and analysis of viroids in 2009 [205]. NGS can provide sequence information on the multitudes of the viroid-derived sRNAs in infected plant materials. Thus, depending on the abundance of vd-sRNAs the molecular fragments of the viroid can be assembled. Due to the small size of the vd-sRNAs (21–24 nucleotides), their sequences can be directly used as primer sequences in RT-PCR amplification of viroid fragments. Detection of plant infections of unknown etiology could be attributed to viroids among other plant pathogens. Two new viroids, the persimmon viroid 2 [206] and GLVd [32] were discovered using NGS. Additionally, the infections of fig and hops trees with the apple dimple fruit viroid (ADFVd) [207] and CBCVd [208], respectively, were discovered using NGS and this showed their wide host range. Table 4 lists some of the viroids and their variants detected by NGS techniques.

Importantly, NGS has become a powerful tool for viroid detection and amplification without prior knowledge of the viroid nucleotide sequence. NGS affords a quick, accurate and sensitive detection technique for identifying viroids affecting several host species and even detecting infections of woody perennials having low titers of these pathogenic organisms. Thus, the detection of newer viroids and their variants has become possible while helping to extend the host range of these viroids. NGS-based diagnosis of viroids has become important for quarantine and certification schemes in addition to operations aimed at elimination of viroid contamination from vegetatively propagated plant material [210,211,212,213]. This makes NGS an efficiently powerful tool to control and contain viroid diseases. If the NGS technology were used in combination with genome engineering methods such as the CRISPR/cas9 system, this will enable better control of these viroids [214,215,216].

Plant pathology necessitates the practice of Koch’s postulates wherein a given RNA molecule with the physicochemical characteristics of a viroid can be cloned and properly assessed in terms of its ability to cause infection upon inoculation and thus determine its biological activity. Therefore, bioassays will invariably have a pivotal role in viroid research. It is obvious that the biological and molecular assays when used synergistically can provide an efficient means for detection, identification and characterization of viroids.

## 11. Origin and Evolution of Viroids

Viroid evolutionary history was proposed to have started independently and earlier than the evolution of DNA and protein in the original RNA world [217]. It is a unique example to study the complex processes of evolution in an extremely simple biological system where expression of phenotype depends directly on the genotype [218]. Infectious agents such as RNA viruses are considered to be directly related to viroids and it has been hypothesized that viroids are the primitive form of RNA viruses [219]. However, there is an alternative hypothesis which suggests that viroids originated from either nuclear RNAs [219], introns [220,221], transposable elements [222] or mitochondrial retroplasmids, which were dismissed based on investigative evidence. Therefore, as suggested by Diener [223], viroids are better candidates to be considered as “living fossils” of a pre-cellular RNA world than introns.

Viroids are subject to strong selection pressure to maintain the internal base pairing of their secondary structure in order to avoid host defense cellular degradation by RNA silencing or plant nucleases. The secondary structure of the viroid RNA is a classic example of an optimal compromise between the essential need for survival against the host defense mechanism and the requirement to recruit host cellular network for replication and transfer [14,224].

Wassenegger and his team [225] demonstrated that a single nucleotide change within the viroid genome can affect its ability to infect the host. This generates a closely resembling sequence variants of viroids exhibiting different host invading properties. On the other hand, some of the plants become host to a number of viroids of distinct genera and even get infected with a mixture of viroids and viruses, which facilitates the swift evolution of viroids [19]. Experiments of site-directed mutagenesis resulted into different disease outcomes indicating that when viroids are exposed to selective pressures, extremely rapid evolution is observed. Under stressful environments for one viroid species, another more suitable and fit quasispecies with variant nucleotide sequence becomes dominant within a very short time period. This extreme capability of genome plasticity distinguishes viroids as the most promptly evolving biological system.

## 12. Future Perspective and Research

Earlier research was directed towards understanding the roles of the structural/functional motifs within the viroid responsible for the induction of viroid-associated symptoms. Then, attention turned towards investigating viroid-host protein interactions. More recently, viroid research has focused on the analysis of the transcriptome of viroid-infected host plants using RNA-seq and other next-generation sequencing technologies and bioinformatics analyses [226]. The viroid-derived small RNA (vd-sRNA) discovered in plants infected with viroids [150] and its role in subverting host gene expression through RNA silencing have implicated the involvement of the vd-sRNA in the expression of viroid-induced disease symptoms. This vd-sRNA-mediated sequence-specific modulation of host gene expression could explain why even a minor sequence change between viroid strains could elicit a wide array of symptoms in susceptible plant cultivars. This calls for further research to explain the molecular mechanism(s) behind the extensive genome degradation seen in the viroid-infected host plants.

Viroids have also been implicated in affecting the host translational machinery. Investigation of the polysome fractions of tomato plants infected with CEVd or PSTVd have identified the presence of the viroid molecule within the ribosomal fraction which show the involvement of viroids in inducing ribosomal stress by direct intervention of the plant translational machinery [143]. However, which viroid regions are involved in such translational interference is yet to be identified. Advancements such as deep sequencing of the extracted polysomes in viroid-infected plants are called for. This could shed further light into our knowledge of viroid–host interactions and their role in determining viroid pathogenicity.

In vitro molecular modeling using thermodynamic folding-based structure predictions as well as direct visualization of the viroid genome by atomic force microscopy are increasingly employed to identify the most stable viroid structure occurring within the cell [227]. This will shed light into the how the mature viroids escape the RNA silencing machinery of their hosts despite triggering the silencing of the host genes. Evidently, the past five decades of viroid research have led to better discernment of viroid structure, molecular biology, pathogenicity and viroid-host interactions. The recent interest in research on coding and noncoding circular RNAs in mammalian cells [228] and their respective functions has resulted in implicating the roles of circular RNAs in diseases such as cancer in humans. Further introspection into the molecular nature and impact of viroids could reveal valuable insights into the nature of circular RNAs in systems other than plants. Thus, viroids despite being minuscule and simple in terms of genomic composition and structure are far more complex than was originally envisioned.

## 13. Conclusions

The literature described above demonstrates that viroid research is flourishing and has uncovered novel methods to characterize various population structures of viroids and how they undergo replication, movement, plant-to-plant transmission and perform host-specific interactions. Considering the appearance of new and recombinant viroids, there is a compelling need to evolve more efficient detection and identification techniques on a continual basis. This will definitely help in the discovery of new intervention techniques to preclude viroid replication and movement and ultimately quell/control viroid-induced diseases.

## Figures and Tables

**Table 1:** Molecular features of viroids considered for classification.

Molecular Characteristics	Family *Avsunviroidae*	Family *Pospiviroidae*
Viroid structure	Y-shaped or branched	Rod-like
Structural/Functional domains	Not applicable	Terminal left (TL), Pathogenicity (P), Central (C), Variable (V) and Terminal right (TR)
Ribozyme function	Yes	No
Site of replication within the host	Chloroplast	Nucleolus
Mode of replication	Symmetric rolling circle mechanism	Asymmetric rolling circle mechanism
Catalytic enzymes	RNA-templated RNA transcription Nuclear-encoded chloroplastic RNA polymerase	RNA-templated RNA transcription DNA-dependent RNA polymerase II
Host plant range	Narrow	Narrow or broad based on viroid species

(Adapted from Adkar-Purushothama and Perreault, 2019 [2]).

**Table 2:** Classification of viroids and viroid-like RNAs. Type member of each genus is indicated in bold. Viroid and viroid-like RNAs unassigned are represented by * symbol. (Adapted from Adkar-Purushothama and Perreault, 2019 [2]).

Family	Genus	Species
Avsunviroidae	Avsunviroid	**Avocado sun blotch viroid**
	Pelamoviroid	**Peach latent mosaic viroid** Chrysanthemum chlorotic mottle viroid
	Elaviroid	**Eggplant latent viroid** Grapevine hammerhead viroid-like RNA * Apple hammerhead viroid-like RNA *
Pospiviroidae	Pospiviroid	**Potato spindle tuber viroid** Tomato apical stunt viroid Tomato chlorotic dwarf viroid Tomato planta macho viroid Columnea latent viroid Citrus exocortis viroid Chrysanthemum stunt viroid Pepper chat fruit viroid Iresine viroid I Portulaca latent viroid *
	Hostuviroid	**Hop stunt viroid** Dahlia latent viroid
	Cocadviroid	**Coconut cadang-cadang viroid** Coconut tinangaja viroid Citrus bark cracking viroid Hop latent viroid
	Apscaviroid	**Apple scar skin viroid** Apple dimple fruit viroid Pear blister canker viroid Citrus bent leaf viroid Citrus dwarfing viroid Citrus viroid V Citrus viroid VI Citrus viroid OS Australian grapevine viroid Grapevine yellow speckle viroid 1 Grapevine yellow speckle viroid 2 Apple fruit crinkle viroid * Grapevine yellow speckle viroid 3 * Grapevine latent viroid * Persimmon latent viroid * Persimmon viroid 2 *
	Coleviroid	**Coleus blumei viroid 1** Coleus blumei viroid 2 Coleus blumei viroid 3 Coleus blumei viroid 4 * Coleus blumei viroid 5 * Coleus blumei viroid 6 *

**Table 3:** Viroid species for which RT-LAMP protocols were developed. (Adapted from Panno et al. 2020 [197]).

Species	Acronym	Family	Genus	Reference
Apple scar skin viroid	ASSVd	Pospiviroidae	Apscaviroid	[198]
Chrysanthemum chlorotic mottle viroid	CChMVd	Avsunviroidae	Pelamoviroid	[191]
Chrysanthemum stunt viroid	CSVd	Pospiviroidae	Pospiviroid	[199]
Coconut cadang-cadang viroid	CCCVd	Pospiviroidae	Cocadviroid	[192]
Columnea latent viroid	CLVd	Pospiviroidae	Pospiviroid	[200]
Peach latent mosaic viroid	PLMVd	Avsunviroidae	Pelamoviroid	[193]
Pepper chat fruit viroid	PCFVd	Pospiviroidae	Pospiviroid	[201]
Potato spindle tuber viroid	PSTVd	Pospiviroidae	Pospiviroid	[194]

**Table 4:** Examples of novel viroids and variants discovered by next-generation sequencing (NGS) technologies. (Adapted from Hadidi, 2019) [12].

Viroid	Description	Reference Source
Persimmon viroid-2	A novel apscaviroid	Ito et al. 2013 [206]
Grapevine latent viroid	A novel apscaviroid	Zhang et al. 2014 [32]
Apple dimple fruit viroid	A novel variant that naturally infects fig	Chiumenti et al. 2014 [207]
CBCVd	A novel variant that naturally infects hops Two novel citrus variants closely related to hop variants	Jakse et al. 2015 [208] Wang et al. 2018 [209]

## Data Availability

Not applicable.
